# An Exploratory Study Using Cortisol to Describe the Response of Incarcerated Women IPV Survivors to MAMBRA Intervention

**DOI:** 10.1155/2016/7068528

**Published:** 2016-09-08

**Authors:** Janette Y. Taylor, Ezra C. Holston

**Affiliations:** ^1^University of Iowa College of Nursing and College of Liberal Arts, Department of Gender, Women's and Sexuality Studies, 50 Newton Road, 474 CNB, Iowa City, IA 52242, USA; ^2^University of Tennessee, 1200 Volunteer Blvd, Rm 353, Knoxville, TN 37996, USA

## Abstract

*Objective*. To determine if incarcerated women survivors of IPV had a physiological response to the Music and Account-Making for Behavioral-Related Adaptation (MAMBRA) intervention, as measured by cortisol levels.* Methods*. A single-group repeated measures designed exploratory study was used to pilot-test MAMBRA. A convenience sample (*n* = 33) was recruited in a Midwestern women's correctional facility. Serving as their own control, participants provided demographics and pre-/post-MAMBRA salivary samples while attending four MAMBRA sessions. Baseline data were compared to participants' data collected over the remaining 3 MAMBRA sessions. Data were analyzed with descriptive and univariate statistics with an alpha of .05 and post-hoc power of .65.* Results*. Participants were predominantly White (52%), single (80%), and early middle-aged (${\overset{-}{x}}_{AGE}=38.7\pm 9.4$), with a history of physical/nonphysical spousal abuse. Using a subsample (*n* = 26), salivary cortisol decreased between the pre-/post-MAMBRA over the sessions (*F*(3,75) = 4.59, *p* < .01).* Conclusion*. Participants had a physiological response to the MAMBRA intervention as evidenced by the decreased cortisol between the pre-/post-MAMBRA. This is the first step in examining MAMBRA's clinical utility as an intervention for female IPV survivors. Future longitudinal studies will examine MAMBRA's effectiveness given this change in cortisol.

## 1. Introduction

Intimate partner violence (IPV) is a preventable public health problem experienced by girls and women across all ages, ethnicities, and socioeconomic levels worldwide [1, 2]. In the United States alone, at least 1 in every 3 women are victims of IPV, experiencing physical abuse, rape, or psychological abuse [3]. Defined as a pattern of assaultive and coercive behaviors, by a current or former intimate partner, IPV may include psychological and physical aggression, intentional physical injury, psychological abuse, sexual assault, progressive social isolation, stalking, deprivation, intimidation, and threats [3]. Witnessing and experiencing IPV can significantly damage women's life course and health. Survivors of IPV often engage in unhealthy behavioral patterns such as self-harm, suicidal ideation, substance abuse, and sexual risk-taking. For some women, IPV is a pathway to illegal activities and incarceration [4–7]. Population-based surveys, done at state and federal prisons, estimate that 43–57% of female inmates have been physically or sexually abused at some time in their lives [8, 9]. Female offenders are 7 times more likely to have experienced sexual abuse and 4 times more likely to report experiencing physical abuse prior to incarceration as compared with male offenders [10]. Various forms and levels of IPV are significant sources of physical trauma as well as acute and chronic stress that can result in physiologic changes such as altered levels of cortisol.

Cortisol is a physiologic measure of the hypothalamic pituitary adrenal (HPA) axis activity. As a physiological marker, altered cortisol indicates a stress response with immediate and long-term health consequences [11–14]. Dysregulation of the HPA axis has been detected in IPV victims and survivors [15, 16]. High levels of cortisol have been reported with the responses of IPV victim-survivors to interpersonal conflict and stimuli such as issues of safety, depressive thoughts, and memories [17, 18]. Chronic and untreated exposure to these IPV-related experiences and events cause further dysregulation of the HPA axis and insufficient cortisol secretion to address the stress responses [16]. These altered cortisol levels have been associated with major depression, posttraumatic stress disorder, suicide, stress, and physical signs of traumatic events, indicating a physiological response to psychological and physical symptoms of IPV [15, 16]. As the symptoms escalate, IPV victims and survivors may have increased suicidal ideation, suicide attempts, and successful suicides [11–14].

Clearly, cortisol has been associated with the stress responses to both psychological and physiological symptoms of IPV. Monitoring the cortisol levels for IPV survivors can be foundational in understanding and treating the personal and interpersonal conflicts that are triggered from the emotional turmoil of IPV [11–18]. Furthermore, routinely assessing the cortisol response may provide a useful way to understand the chronic conditions that result from IPV. However, it is important to take the first critical step in examining the utility of cortisol in the clinical application of trauma informed and gender sensitive interventions. This indispensable first step is to initially determine if the IPV survivors have a physiological reaction to an intervention such as MAMBRA.

This needed first step is further evident because most physiological symptoms are often treated with pharmaceutical and psychotherapeutic interventions that do not address the psychoeducational need of women, especially incarcerated women. Another notable absence in the literature is the research indicating that there is a physiological response of incarcerated women IPV survivors to gender sensitive and trauma informed interventions that address their psychoeducational needs. Therefore, it remains unknown if a physiologic measure such as cortisol can be used to measure a physiological reaction to appropriate interventions. Moreover, despite the large numbers of incarcerated women who have experienced IPV, studies of women in prisons and jails, as a distinct and separate population, have been numerically limited [6, 19, 20]. The research question was “can the physiological response of incarcerated IPV survivors be measured by a change in cortisol levels after engaging in MAMBRA sessions?” Thus, the objective of this repeated measures designed exploratory study was to determine if incarcerated women survivors of IPV had a physiological response to the Music and Account-Making for Behavioral-Related Adaptation (MAMBRA) intervention. In other words, this exploratory study was looking to see if the physiological response was a decrease, increase, or no change in the cortisol levels. MAMBRA, a gender sensitive and trauma informed intervention, includes music, psychoeducation, and account-making (i.e., storytelling).

## 2. Materials and Methods

### 2.1. Participants and Setting

This cortisol data is from the same patient population and intervention as previously published. In that study we assessed the impact of MAMBRA on the psychological symptoms of incarcerated women with histories of abuse [21]. We are reporting the cortisol findings separately because the purpose of the parent study was to separately describe the participants' psychological and physiological responses to the MAMBRA intervention.

This exploratory study used a single-group repeated measures design to pilot test the MAMBRA intervention. A convenience sample of 33 inmates was recruited in a women's correctional facility in the Midwest, the Iowa Correctional Institution for Women. The mean age was 38.7 (± 9.4) at baseline (BL). All participants reported a past lifetime history of IPV. They had to be free of any abusive relationships for at least 1 year to participate in the study. Recruitment occurred through the use of flyers, a noncoercive recruitment strategy, that provided a detailed description of the study. To be eligible, participants had to read and speak English, be at least 21 years of age, and free of abusive relationships for at least 1 year. At the first or BL session, the sample consisted of 33 female participants: over the 4 sessions, 26 participants provided salivary samples that yielded measurable cortisol levels. All participants signed an informed consent document. The study protocol was approved by the Iowa Correctional Institution for Women and the Institutional Review Board at the University of Iowa.

### 2.2. Description and Procedure of the MAMBRA Intervention

Music and Account-Making for Behavioral-Related Adaptation (MAMBRA) is a group-based intervention that uses Participatory Compact Disc (CD) Design and psychoeducation to encourage IPV survivors to explore their experiences of violence and recovery. Participatory CD Design includes the interactive use of CD listening to encourage participants' narratives and discussion [28]. Listening to prerecorded music is a “receptive experience” when clients listen and respond verbally [29, page 48]. The use of music for healing and recovery is an internationally recognized practice used by therapist and informal groups worldwide [30, 31]. Women's music defined as “music by women, for women, and about women” is usually employed to enhance the learning process because of the increased understanding of women's lives and their relationships to broader social conditions [32, page 242]. Women's genre and music lyrics can be evaluated to capture the target audiences' cultural values and beliefs for violence-free living. Among female incarcerated IPV victim survivors, women's music prompted participants to consider how to use music for self-expression and self-care of symptoms related to their experiences with IPV [21]. For this exploratory study, the number of sessions needed to address the women's immediate psychoeducation concerns were 4 sessions. However, the number of sessions can expand to include as many sessions and topics as defined by women. As a result, MAMBRA is a gender sensitive and trauma informed psychoeducation intervention. Additionally, this intervention with its use of music is generally of low cost and easily accessible to women across a range of treatments and settings. It facilitates women's ability to assume responsibility and participation in their recovery.

Participants attended MAMBRA sessions according to their assigned units (e.g., general population, minimum live-out, and substance abuse treatment units) in compliance with the prison policy of prohibiting interaction among some prison units of incarcerated women. The first author facilitated the sessions during the afternoon (6–8 pm). The MAMBRA intervention was administered over 4 sessions. The sessions were scheduled for every two weeks over a 2-month period with each session lasting 1–1.5 hours. For 15–20 minutes, each session had a psychoeducation presentation about a topic related to IPV and recovery. Next, participants listened to and viewed a printed copy of the lyrics for the 1 or 2 music selections whose themes supported the presented psychoeducation topic. After the music selection(s) ended, participants discussed the topic and related it to the music as well as their experiences. Specific CD music was used across MAMBRA sessions with Dianne Reeves' “Testify” [27] played at the end of each session (see Table 1). For the opening session (Session 1), participants listened to Eryka Badu's “Tyrone” [22] and afterwards reviewed dynamics of IPV using the Duluth Model Power and Control Wheel. Participants also identified themes in the lyrics and their lives. In Session 2, an overview of consequences and symptoms of IPV was presented. Women engaged in comparative analysis of their symptoms and theme presented in the lyrics of Kelly Clarkson's “Because of You” [23] and Martina McBride's “A Broken Wing” [24]. Session 3 consisted of an education component centered on social and self-definition of womanhood and Koko Taylor's “I'm A Woman” [25]. Immediately afterwards, participants discussed the roles they valued and why these roles were important to them. During session 4, psychoeducation and the discussion focused on creating and valuing healthy relationships with adult partners and children (e.g., mothering and motherhood). Alicia Keys' “A Woman's Worth” [26] was used for thematic analysis by the women. As the session-closing song, “Testify” [27] was appropriate because it expresses how unexpected and often difficult experiences of life contribute to our becoming the people we are today. The song speaks to the healing potential of testimony and its power to bring us to a fuller understanding of life experiences. Participants were introspective and agreed with the artist's message.

### 2.3. Instrument

After a review of the literature, the Index of Spouse Abuse (ISA) was selected to assess for the occurrence of IPV [33]. The ISA has been used in research with female IPV survivors who were incarcerated and/or African American. The ISA is a 30-item self-administered 5-point Likert scale that measures physical (ISA-P) and nonphysical (ISA-NP) abuse perpetrated against women by an intimate partner. The ISA-P and the ISA-NP are two 15-item subscales of ISA. Initially, ISA-P and ISA-NP internal consistency reliabilities were reported as being above .90 [33]. More recently, the ISA had been successfully used in a women's prison setting with a Cronbach *α* of .98 [34]. The internal consistency reliabilities of ISA-P and ISA-NP in African American women were found to be over .90 [35, 36].

Demographic data were assessed with a PI-generated survey. Demographic data included information about age, race/ethnicity, marital status, education/educational level, income, health status, and prescribed medication and drug use.

### 2.4. Cortisol Analysis

Salivary cortisol was selected as a relatively low risk invasive physiological measure that met prison inspection and in which participants could collect samples and engage in the research. Several minutes before beginning each session, participants were given precoded/prelabeled Salivette vials for saliva collection. Over the session's time frame, samples were collected before the session (pre-MAMBRA) and at the end of the session (post-MAMBRA). It is a known fact that cortisol's decreasing diurnal slope occurs over several 2 to 3-hour intervals, indicating minimal diurnal variation between a pre-/postcortisol measure within one of the 2 to 3-hour intervals. Saliva samples were assessed for free cortisol levels using the HS-Cortisol High Sensitivity Salivary Cortisol Enzyme Immunoassay Kit [37]. “Studies consistently report high correlations between serum and salivary cortisol, indicating that salivary cortisol levels reliably estimate serum cortisol levels” [37, page 1].

### 2.5. Data Analysis

Data were analyzed with SPSS 23.0 (Windows). The data consisted of demographics (age, race/ethnicity, education level, marital status, income, health status, substance abuse treatment, and use of prescribed medications) and cortisol levels. Participants were their own control over the 4 sessions. Participants' baseline data were compared to their data collected over the remaining 3 MAMBRA sessions. At baseline (BL), descriptive statistics were used to characterize the sample and identify outliers and missing data. The cortisol data did not initially have a normal distribution (Kolmogorov-Smirnov statistics = .84–.32, *p* = .002). It was skewed to the right. With log_10_ transformation, the cortisol data had a normal distribution with a normal curve (Kolmogorov-Smirnov statistics = .09–.17, *p* = .58–.20) without altering the measured cortisol. There were 208 data points for analysis (26 participants × 4 sessions × 2 cortisol collections). Single-group repeated measures ANOVA was used to determine how the cortisol levels changed over the 4 MAMBRA sessions. A subsample (*n* = 26; 79% of entire sample) was used after addressing outliers and missing data points identified in the descriptive statistics. Calculating the cortisol mean created a guideline to identify cortisol levels that were outliers (>3 standard deviations from the mean) and to adjust for any fluctuations in cortisol levels due to the collection time of the salivary samples. Data were not included for participants with outlier values >3 standard deviations from the cortisol mean (*n* = 2), missing 5 out of the 8 cortisol data points (*n* = 2), or with no reported cortisol data (*n* = 3). For 5 participants, missing cortisol data were addressed by using either a computed mean from the available cortisol data or one of the available Salimetrics cortisol assay results. Salimetrics provided two cortisol assays for each sample and computed the mean. Salimetrics used a reliable procedure for the assays, and there was no statistical difference between the two cortisol assays. The level of significance was .05 with a post-hoc power of .65 and a small effect size of .20 (G^*∗*^Power) [38].

## 3. Results

At BL, the sample (*n* = 33) consisted of incarcerated women with a mean age of 38.7 (± 9.4) and a history of both physical and nonphysical spousal abuse. Fifty-two percent (*n* = 17) self-reported White, with 76% (*n* = 25) single (not with a partner), 82% (*n* = 27) having at least a high school education, and 80% (*n* = 20) a household income less than $10,000 (see Table 2). Ninety-four percent (*n* = 29) reported the use of street drugs with 76% (*n* = 25) reporting a history of substance abuse treatment. Sixty-one percent (*n* = 20) self-rated their health as good or better with 89% (*n* = 25) on prescribed medications.

**Figure 1:**
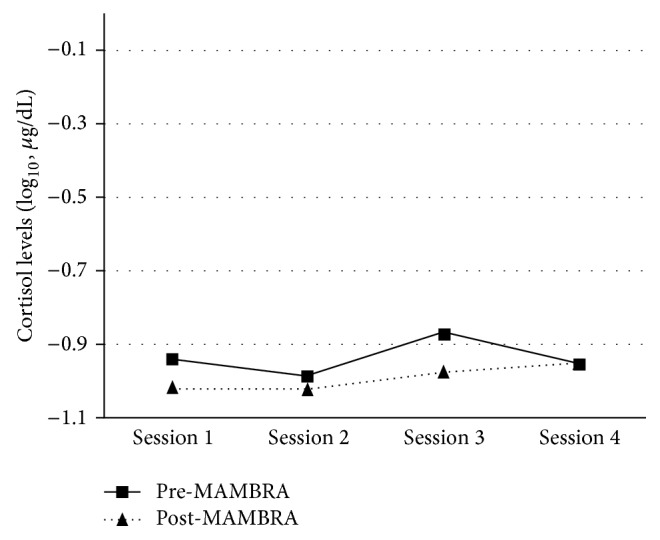
The pre-/post-MAMBRA cortisol levels across the 4 sessions.

Single-group repeated measures ANOVA was used with the subsample of 26 participants. The pre-MAMBRA mean scores were higher than those for post-MAMBRA in all sessions except for the 4th (see Figure 1). In the 4th MAMBRA session, the pre-MAMBRA cortisol was the same as the post-MAMBRA. The 3rd MAMBRA session had the greatest change between the pre-/postcortisol levels. There was a significant main effect in that the post-MAMBRA cortisol levels decreased from the pre-MAMBRA (*F*(1,25) = 5.13, *p* = .03). A significant interaction occurred between the cortisol levels and the MAMBRA sessions (*F*(3,75) = 4.59, *p* < .01). As shown in Figure 1, the pre-MAMBRA cortisol is higher within each session and across the first 3 sessions. Specifically, session 1 pre-MAMBRA cortisol was over .04 log_10_⁡ *μ*g/dL higher than post-MAMBRA cortisol of sessions 1, 2, and 3. However, session 1 pre-MAMBRA cortisol was only .01 log_10_⁡ *μ*g/dL higher than the pre- and post-MAMBRA cortisol of session 4. Given these results, participants had a more pronounced physiological response to the MAMBRA intervention over sessions 1–3 and a slight physiological response between sessions 1 and 4, as measured by the cortisol change. Further examination of this occurrence is warranted in future research.

## 4. Discussion

To pilot-test the MAMBRA intervention, this exploratory study used salivary cortisol as an objective measure of the female incarcerated IPV survivors' response to MAMBRA. A change in cortisol is related to the difference between pre-/post-MAMBRA cortisol levels within the intervention sessions. To the best of our knowledge, this is the first time salivary cortisol levels have been used to describe the physiological response of female incarcerated IPV survivors to a gender sensitive, trauma informed, and psychoeducation intervention (e.g., MAMBRA). This study is also one of the first to describe how the cortisol changed within and across the MAMBRA sessions. Specifically, the post-MAMBRA cortisol levels were lower than the pre-MAMBRA within sessions 1–3. Interestingly, the session with the greatest cortisol change (session 3) focused on participants discussing their definition of womanhood, their roles as women, and why they valued these roles. Perhaps this session generated the most insight and positive thinking, which should be investigated in future studies. Nevertheless, the results of this study indicate that participants had a physiological response to the MAMBRA intervention that was objectively measured with the salivary cortisol.

Our findings are similar to those reported by other researchers using a variety of interventions tailored to the participants' needs. Jones et al. [39] examined the cortisol levels in 4 sessions using specific relaxation techniques with HIV seropositive women. Cortisol levels significantly decreased in two sessions (*p* ≤ .01) and the remaining two approached significance (*p* ≤ .09). The greatest cortisol decrease occurred in the session using guided imagery tailored to the participants' specific needs. Bershadsky et al. [40] examined how cortisol levels changed during Hatha yoga for 2 groups of pregnant women (yoga-engaging versus non-yoga engaging). The yoga days had a significant decrease in cortisol level (*p* = .01) for the early-pregnant and mid-pregnant participants. The cortisol level for the yoga-engaging pregnant participants was significantly less than the non-yoga-engaging pregnant participants (*p* = .03). Cortisol levels significantly decreased with an intervention specific to the participants' needs. These findings parallel our findings since their participants had a decrease in the cortisol after an intervention specific to their needs.

In this study, the physiologic response of the incarcerated women as measured by salivary cortisol corresponded with previous studies that used only music therapy. After inducing mental fatigue (intelligence and social stress tests) in healthy participants, Khalfa et al. [41] and Suda et al. [42] found a significant decrease in salivary cortisol levels (*p* = .02 and *p* < .01, resp.) among music listeners compared to those in a silent control condition. Both studies indicated that mentally fatigued participants were responsive to music as demonstrated by the lowering of their elevated cortisol levels. Similarly, Nakayama et al. [43] reported a lowering of salivary cortisol (*p* = .03) in an effectiveness study of music therapy with 10 patients in hospice care. Chen et al. [44] also reported a significant reduction in salivary cortisol levels, over time, as a result of Chinese five-element music therapy among 31 depressed nursing students randomly assigned to a music group. These studies suggest that the response to the relaxing and meaningful music therapy can be assessed by the decreasing cortisol levels.

Thus, the overall conclusions from the above studies resonate with the conceptualization of MAMBRA. Through a combination of psychoeducation and participatory CD Design, MAMBRA is created to meet the needs of female IPV survivors, especially those who are incarcerated. Furthermore, the physiological response as measured by cortisol indicates the reactivity of incarcerated women to MAMBRA; perhaps this suggest that MAMBRA may facilitate women's responsiveness about their IPV experiences and symptoms.

Activities such as psychoeducation and group discussion help female inmates to formulate accounts that cope with the psychological effects of IPV (e.g., depression, anxiety, self-esteem, and social isolation) [21]. With music in therapy, a therapeutic mediator is established that increases social interaction, improves quality of life, enhances personal growth, and increases self-actualization [45]. MAMBRA (an account-making, music therapy, and group support intervention) collectively promotes identity or self-concept and facilitates symptom management. Additionally, gender and culture are central to how individuals define, perceive, label, explain, and evaluate personal health experiences [46, 47]. MAMBRA addresses issues specific to the women's culture and racial/ethnic background in the context of violence and abuse. As such, it is a gender sensitive and trauma informed group therapy intervention that provides opportunities for women (especially incarcerated women) to learn and improve upon adaptive behaviors (i.e., prosocial action for themselves, their families, and communities) [48, 49]. MAMBRA also promotes healing, mental health, and self-management of symptoms related to past IPV, which are needed for female incarcerated IPV survivors who will at some point struggle to reintegrate back into society [21].

Although this study focused on using cortisol as an objective measure of the physiologic response to MAMBRA, we noted that the cortisol levels were high prior to each session. An alternative hypothesis for this high cortisol level could be situational events related to the prison setting in which the women resided. Additionally, this elevated pre-MAMBRA cortisol could be related to anticipation about confronting feelings that may surface during the MAMBRA intervention. Nevertheless, high cortisol levels decreased immediately after MAMBRA, which indicated the participants' physiological response to MAMBRA. Further investigation of this change in cortisol levels is warranted because it may possibly be a short-term effect. Perhaps this change might be useful in discussing the no change in the pre- and post-MAMBRA cortisol levels for session 4. The high pre-MAMBRA cortisol levels may have persisted because the topics in session 4 dealt with issues that were not in the participant's immediate future or interest. These women may have felt a level of futility in discussing the possibility of resuming their roles as mothers and intimate partners within a violent-free living condition. They are still incarcerated and must deal with issues related to parenting from the prison setting. Nevertheless, the pattern of high pre-MAMBRA cortisol levels changing to low post-MAMBRA cortisol levels of sessions 1–3 warrants further investigation in future studies about the effectiveness of MAMBRA.

In our study, each psychoeducation topic is built on the other so that MAMBRA created a therapeutic environment. As evidenced by the change in cortisol, this structure may have allowed participants to comfortably think about and physiologically response to symptoms of IPV and dormant feelings through reappraisal [50, 51]. As dormant feelings emerged, participants may begin to process subjective feelings associated with IPV. Participant's response to this emergence may have been measured by the slight increase in the post-MAMBRA cortisol level from the first session to the last. This finding may show that a physiologic response to MAMBRA may indicate that some dormant feelings are beginning to emerge, allowing patients to think about other feelings that were not identified. As Saxena et al. [10] suggested, some women may not have defined their abuse experiences as trauma initially. However, participation in MAMBRA will likely facilitate their ability to conceptualize, understand, and accept the abuse as a traumatic experience, thus resulting in a response.

This study initializes the process to demonstrate that MAMBRA can establish an environment that may enhance the treatment of women. There are several implications for practice by healthcare providers and advocates in prison setting, community-based correction facilities, and community services. By utilizing a gender sensitive and trauma informed music invention, health care providers and advocates can more actively engage and address women's psychoeducational needs. With MAMBRA, healthcare providers in prison settings, community-based correction facilities, and shelters can enhance the healing potential for female survivors of IPV through open dialog, self-reflection, insight, and importantly the ability to talk about abuse within the family while processing family dynamics. Furthermore, MAMBRA may be useful in helping female survivors of IPV empower themselves and regain autonomy, especially for women in male-dominant cultures or societies that accept abusive behavior towards women as normal.

Most importantly, having an objective measure of an intervention such as MAMBRA could facilitate the implementation of the WHO recommendations in treating IPV and sexual violence against women [2]. Internationally, the female prison population is growing on all five continents [52]. Many of these women have encountered violence in their lives and often suffered silently from the aftermath. MAMBRA is an intervention that could be adapted for incarcerated women worldwide and across a variety of treatment options. Because our study focused on incarcerated female IPV survivors in the U. S., additional research is needed to examine the intervention and its various applications for international practice.

There were 3 limitations of this exploratory study. First, there was researcher bias because the intervention was facilitated by the first author. The first author planned and discussed the sessions with the research team (the second author and a graduate student) in an attempt to minimize this bias. Second, a convenience sampling was recruited within the prison setting, decreasing the generalizability of the findings. This limitation was addressed by performing statistical analyses with data representative of a consistent response across the MAMBRA sessions. Finally, the sample size was small. This third limitation was addressed by determining the post-hoc power. While complying with prison protocol and policies, future studies should recruit a larger sample by using multiple data collection sites and better integrate the intervention schedule with established prison routines and schedules. Also, the participants can serve as their own control.

## 5. Conclusions 

Cortisol levels in incarcerated victims of IPV decreased from the beginning of the MAMBRA session to the end, indicating participants' physiologic response to the MAMBRA intervention. This physiologic reactivity is the first step in determining if incarcerated female IPV survivors would respond to MAMBRA and in establishing an objective measure of an intervention to address the psychoeducational needs of female IPV survivors for better management of symptoms related to IPV. Our study's results initiate an understanding for the potential role of cortisol as an additional measurement in the clinical care of incarcerated women who have been victimized by IPV.

## Figures and Tables

**Table 1:** Song selection for MAMBRA sessions.

Song	Session	Rationale
Eryka Badu's *Tyrone* [22]	1	To review dynamics of IPV

Kelly Clarkson's *Because of You* [23]	2	To engage women in comparative analysis of their symptoms and those presented in the lyrics
Martina McBride's *A Broken Wing* [24]		

Koko Taylor's *I'm a Woman* [25]	3	To facilitate women's self-definition of woman and to challenge long held assumptions of gender identity and roles

Alicia Keys' *A Woman's Worth* [26]	4	To think about focal points for healthy relationships with self, adult partners, and children (e.g., mothering and motherhood)

Dianne Reeves' *Testify* [27]	1, 2, 3, and 4	To encourage women to engage in the often difficult, but healing, process of bearing witness through oral testimony

**Table 2:** Demographics.

	Sample (*n* = 33)	Subsample (*n* = 26)
Mean age	38.7 ± 9.4	39.0 ± 9.7

Mean ISA-P	62.3 ± 20.7	62.5 ± 21.0

Mean ISA-NP	64.5 ± 16.9	64.6 ± 17.2

Race/ethnicity		
African American	13 (39%)	8 (31%)
White	17 (52%)	16 (61%)
Biracial	2 (6%)	1 (4%)
Hispanic	1 (3%)	1 (4%)

Marital status		
Single	15 (46%)	12 (46%)
Married	5 (15%)	4 (15%)
Divorced	7 (21%)	5 (19%)
Separated	3 (9%)	3 (12)
Unmarried couple	3 (9%)	2 (8%)

Highest education		
<12th grade	6 (18%)	4 (15%)
High school/GED	10 (30%)	9 (35%)
Some college	14 (43%)	11 (42%)
2-year college degree	3 (9%)	2 (8%)

Household income		
<$10,000	20 (80%)	17 (81%)
<$20,000	2 (8%)	2 (9%)
<$30,000	1 (4%)	1 (5%)
<$60,000	1 (4%)	
Unknown	1 (4%)	1 (5%)

Health status		
Poor/fair	13 (39.5%)	12 (46%)
Good	8 (24%)	7 (27%)
Very good/excellent	12 (36.5%)	7 (27%)

Use prescribed meds, yes	25 (89%)	19 (86%)

Use street drugs, yes	29 (94%)	23 (96%)

HX S-abuse TX, yes	25 (76%)	20 (77%)

Note: ISA-NP = index of spouse abuse-nonphysical; ISA-P = index of spouse abuse-physical; HX = history; TX = treatment.
